# A Diastereomeric Hydroxycurvularin Mixture Inhibits Melanogenesis and Inflammation *via* Modulation of Tyrosinase Activity and Cytokine Expression

**DOI:** 10.4014/jmb.2603.03015

**Published:** 2026-07-02

**Authors:** Ja-Hun Seo, Hyun-Su Koo, Van-Hieu Mai, Su-Bin Kim, Gi-Hyeon Seong, Thuong T. T. Nguyen, Jeong-Eun Seon, Ha-Rim Choi, Hyang Burm Lee, Won-Keun Oh, Hyung-Sik Kang

**Affiliations:** 1 School of Biological Sciences and Technology, Chonnam National University, Gwangju 61186, Republic of Korea; 2 Research Institute of Pharmaceutical Sciences, College of Pharmacy, Seoul National University, Seoul 08826, Republic of Korea; 3 Department of Agricultural Biological Chemistry, Chonnam National University, Gwangju 61186, Republic of Korea; 4 Department of Nursing, Nambu University, Gwangju 62271, Republic of Korea

**Keywords:** Fungal polyketides, Hydroxycurvularin, *Curvularia intermedia*, Melanogenesis, Pigmentation regulation, Dermatological potential

## Abstract

Fungal metabolites serve as a prolific source of structurally diverse bioactive compounds with significant potential for skin health and pigmentation regulation. In this study, two diastereomers of 11-hydroxycurvularin (compounds **1** and **2**) and curvularin (compound 3) were isolated from *Curvularia intermedia* and evaluated for their anti-melanogenic and immunomodulatory effects. The diastereomeric mixture of 11-hydroxycurvularins (HC mix) exhibited superior anti-melanogenic activity in α-MSH–stimulated B16F10 melanoma cells, surpassing the efficacy of the individual diastereomers, the parent compound 3, and the positive control arbutin, without inducing cytotoxicity. Mechanistically, the HC mix exerted dual regulatory effects by inhibiting tyrosinase enzymatic activity and downregulating the transcription of core melanogenic genes (Tyr, Tyrp1, and Tyrp2). Furthermore, the HC mix attenuated LPS-induced reactive oxygen species (ROS) production and selectively regulated inflammatory gene expression in RAW 264.7 macrophages and primary murine splenocytes, downregulating IL-1β and IL-6 while upregulating TGF-β. These findings highlight that hydroxyl substitution at C-11 is critical for enhancing biological potency. Collectively, the HC mix represents a potential candidate for managing pigmentary and inflammatory skin disorders, warranting further validation in human models.

## Introduction

Melanin plays a central role in skin homeostasis by acting as an endogenous defense molecule that mitigates environmental stress by scavenging reactive oxygen species (ROS), absorbing ultraviolet (UV) radiation, and forming a barrier against microbial invasion [1, 2]. Chronic exposure to UV radiation, oxidative stress, and pro-inflammatory cytokines can dysregulate melanogenesis, resulting in pathological hyperpigmentation and contributing to skin aging [3]. Given the key roles of oxidative and inflammatory pathways in aberrant melanin production, antioxidant and anti-inflammatory compounds have emerged as promising modulators of melanogenesis [3, 4].

Melanin biosynthesis is initiated by the sequential oxidation of L-tyrosine and L-3,4-dihydroxyphenylalanine (L-DOPA), catalyzed by the rate-limiting enzyme tyrosinase [5, 6]. This enzymatic process is further regulated by tyrosinase-related proteins **1** and **2** (Tyrp1 and Tyrp2), under the transcriptional control of α-melanocyte-stimulating hormone (α-MSH) [5, 7]. Although numerous tyrosinase inhibitors, including hydroquinone, kojic acid, and arbutin, are commercially used in skin-lightening formulations, their clinical application is constrained by limitations in stability, safety, or efficacy [8]. These challenges highlight the need for safer and more efficient melanogenesis inhibitors with multifunctional properties.

To address these limitations, structurally diverse fungal secondary metabolites are increasingly recognized as promising sources of novel depigmenting agents. Both individual compounds and complex mixtures from fungi contribute significantly to the repertoire of bioactive pharmaceuticals and cosmetic ingredients [9]. These metabolites exhibit diverse biological activities, including anti-inflammatory, anticancer, and anti-melanogenic effects [10, 11]. Notably, members of the *Ascomycota* and *Basidiomycota* phyla are known to produce cosmetic ingredients with skin-whitening, moisturizing, and anti-aging activities [12], while *Zygomycota* fungi such as *Rhizopus arrhizus* and *R. oryzae* are utilized industrially to produce organic acids, including lactic acid, a widely used skin-conditioning agent [13].

Among fungal polyketides, curvularin and its derivatives are recognized for their wide-ranging bioactivities. Curvularin, a macrocyclic lactone, was initially isolated from a fungus of the genus *Curvularia* [14] and has since been identified in several ascomycetous fungi, including *Alternaria* [15], *Aspergillus* [16], *Cochliobolus* [17], and *Penicillium* [18]. A marine-derived *Penicillium* strain was previously reported to produce curvularin analogs, including six curvularin derivatives: (11R,15S)- and (11S,15S)-11-hydroxycurvularin, (11R,15S)- and (11S,15S)-11-methoxycurvularin, and (10E,15S)- and (10Z,15S)-10,11-dehydrocurvularin [18]. These macrocyclic lactones exhibit diverse biological functions, including phytotoxic [19], antifungal and antibacterial [20], anti-inflammatory [18], and cytotoxic activities [16]. However, their potential roles in melanogenesis remain underexplored.

In the present study, we identified a fungal strain, CNUFC 23CHB15, isolated from a *Nephila* spider collected in South Korea (36°26′16.2″N, 126°46′04.6″E), which produces curvularin derivatives. Although similar compounds have been associated with antibacterial, anti-inflammatory, and anti-allergic effects, this is the first report demonstrating their inhibitory activity on melanogenesis. Our findings reveal the multifunctionality of hydroxycurvularin-type fungal polyketides, highlighting their potential as bioactive agents for the management of pigmentary and inflammatory skin conditions.

## Materials and Methods

### Sample Collection, Isolation, and Observation of Morphology

A golden orb-weaving spider (*Nephila* sp.) sample was collected at Kunryang-ri, Cheongyang-eup, Cheongyang, Chungnam Province, South Korea (36°26′16.2″N, 126°46′04.6″E), in October 2022. The sample was captured and handled with gloved hands, placed in polyethylene bags, and stored at ambient temperature until transported to the laboratory. Cadavers were broken up into small pieces and placed on potato dextrose agar (PDA, Difco, USA). The plates were then placed in the dark at 25°C and checked under a stereomicroscope every day after the second day of incubation. Then, hyphal tips were transferred to fresh PDA. Pure isolate (CNUFC 23CHB15) was stored in 20% glycerol at -80°C and PDA slant tubes at the Environmental Microbiology Laboratory Fungarium, Chonnam National University (CNUFC), Gwangju, South Korea. The strain was grown in triplicate on PDA, malt extract agar (MEA; 20 g of malt extract and 20 g of agar in 1 L of deionized water), and oatmeal agar (OA; 30 g of oatmeal and 20 g of agar in 1 L of deionized water) and incubated at 25°C in the dark for 14 days. Microstructures were observed under light microscopy (Olympus BX53, Japan) after mounting fragments of the fungal mycelia in 60% lactic acid.

### DNA Extraction, PCR, and Sequencing

Fresh mycelium was scraped from the margins of colonies on PDA overlaid with cellophane and incubated at 25°C for 7 days. Genomic DNA was extracted using SolgTM Genomic DNA Preparation Kits according to the manufacturer’s instructions (Solgent Co. Ltd., Republic of Korea). The purified DNA was stored at -20°C for later use. Three regions of the internal transcribed spacer (ITS) region were amplified, using the primers V9G/ITS 4 (White *et al*. 1990, de Hoog and Gerrits van den Ende 1998); *GAPDH* gene, using the primers gpd1 and gpd2 [21]; and *TEF1* gene, using the primers EF983 and EF2218R [22]. The PCR procedure was carried out in a 20 μL reaction volume mixture containing 2 μL (100–200 ng) of template DNA and 1.5 μL of each primer (10 pmol/μL) using AccuPower PCR PreMix (Bioneer, Republic of Korea). All PCR products were immediately visualized on 1% agarose gels stained with SafeView™ dye (1 μL/10 mL of agarose). The PCR products were purified with Accuprep PCR Purification Kits (Bioneer Corp., Republic of Korea). DNA sequencing was performed at Macrogen (Republic of Korea), using the same primers employed for PCR.

### Phylogenetic Study

The taxa used in the phylogenetic analysis were downloaded from GenBank (www.ncbi.nlm.nih.gov/genbank/). SeqMan v. 7.0.0 (DNAstar, USA) was used to assemble the consensus sequences. The sequences were aligned using the MAFFT v.7 online server (http://mafft.cbrc.jp/alignment/server/) [23] and manually optimized in MEGA v.7 [24]. Maximum-likelihood (ML) analysis was performed using RAxML-HPC2 on XSEDE (v. 8.2.12) via the CIPRES Science Gateway (https://www.phylo.org/portal2), with a GTR model and rapid bootstrap analysis followed by 1,000 bootstrap replicates. The consensus trees were viewed in FigTree v. 1.3.1 [25].

### Fermentation, Extraction, and Isolation

The strain CNUFC 23CHB15 was cultured on potato dextrose agar (PDA; Becton, USA) at 25°С for 5 days. Fungal mycelial plugs were carefully removed using a 6.5 mm diameter cork borer. The plugs were transferred into 20 1 L Erlenmeyer flasks (each flask containing 200 g rice and 200 mL of distilled water, previously sterilized at 121°C for 15 min). Fermentation was performed at 25 °C under static conditions for 3 weeks. After fermentation, fungal cultures were extracted twice with methanol (1 L per flask) at room temperature for 24 h. After filtering, the MeOH extractant was concentrated to yield a brownish slurry of crude extract, which was then suspended in 4 L of water and partitioned against ethyl acetate (3 × 3 L). A portion of the ethyl acetate extract (250 mg) was subjected to medium-pressure liquid chromatography (MPLC) on an RP-C18 column (50 g, Biotage) using a gradient elution of 50–100% MeCN containing 0.1% formic acid, affording 15.5 mg of a mixture of compounds **1** and **2**, 98 mg of compound **3**. The mixture of compounds **1** and **2** (12 mg) was further purified by semi-preparative high-performance liquid chromatography (HPLC) on an OptimaPak C18 column (RS Tech) using a gradient of 45–50% MeOH containing 0.1% formic acid to yield compound **2** (5.8 mg) and compound **1** (4.6 mg), respectively. (11*R*,15*R*)-11-Hydroxycurvularin (**1**): White solid; IR (KBr) ν_max_ 3285, 2921, 2861, 1710, 1611, 1457, 1335, 1276, 1165, 1046, 994, 850, 757, 654 cm^-1^; ^1^H NMR (MeOD-*d4*, 400 MHz) δ 6.28 (1H, d, *J* = 2.3 Hz), 6.21 (1H, d, *J* = 2.3 Hz), 4.98 (1H, m), 4.09 (1H, m), 3.97 (1H, d, *J* = 15.7 Hz), 3.59 (1H, d, *J* = 15.7 Hz), 3.22 (2H, d, *J* = 7.2 Hz), 1.68 (1H, m), 1.62 (1H, m), 1.45 (4H, m), 1.12 (3H, d, *J* = 6.4 Hz); ^13^C NMR (MeOD-*d4*, 100 MHz) δ 205.7, 172.5, 161.9, 160.9, 138.2, 120.0, 112.6, 102.9, 72.3, 68.7, 53.6, 41.2, 34.9, 32.3, 19.2, 19.2; HRESIMS *m/z* 309.1330 [M+H]^+^ (calcd. for C_16_H_20_O_6_, 309.1333). (11*S*,15*R*)-11-Hydroxycurvularin (**2**): White solid; IR (KBr) ν_max_ 3283, 2932, 2861, 1715, 1612, 1459, 1337, 1278, 1163, 1041, 994, 850, 757, 654 cm^-1^; ^1^H NMR (MeOD-*d4*, 400 MHz) δ 6.26 (1H, d, *J* = 2.3 Hz), 6.21 (1H, d, *J* = 2.1 Hz), 4.84 (2H, m), 4.08 (1H, tt, *J* = 7.5, 3.9 Hz), 3.87 (1H, d, *J* = 14.2 Hz), 3.65 (1H, m), 3.59 (0H, m), 2.82 (1H, t, *J* = 10.8 Hz), 1.13 (3H, d, *J* = 6.2 Hz); ^13^C NMR (MeOD-*d4*, 100 MHz) δ 206.6, 172.7, 161.7, 159.9, 137.4, 112.2, 102.8, 74.9, 67.7, 54.9, 40.2, 35.5, 32.6, 23.2, 21.5; HRESIMS *m/z* 309.1337 [M+H]^+^ (calcd. for C_16_H_20_O_6_, 309.1333). Curvularin (**3**): White solid; IR (KBr) ν_max_ 3285, 2930, 2865, 1713, 1608, 1457, 1337, 1278, 1165, 1041, 994, 850, 757, 654 cm^-1^; ^1^H NMR (MeOD-*d4*, 400 MHz) δ 6.25 (1H, d, *J* = 2.3 Hz), 6.22 (1H, d, *J* = 2.3 Hz), 4.90 (2H, m), 3.86 (1H, d, *J* = 15.9 Hz), 3.62 (1H, d, *J* = 15.9 Hz), 3.21 (1H, ddd, *J* = 15.2, 8.9, 2.7 Hz), 2.74 (1H, ddd, *J* = 15.3, 9.7, 2.7 Hz), 1.73 (1H, m), 1.57 (2H, m), 1.44 (2H, m), 1.38 (1H, m), 1.30 (1H, m), 1.24 (1H, m), 1.12 (3H, d, *J* = 6.3 Hz) ; ^13^C NMR (MeOD-*d4*, 100 MHz) δ 209.8, 172.9, 161.3, 159.6, 137.3, 120.9, 112.3, 102.7, 73.8, 49.3, 49.0, 48.8, 44.7, 40.6, 33.0, 27.8, 24.9, 23.9, 20.5; HRESIMS *m/z* 293.1370 [M+H]^+^ (calcd. for C_16_H_20_O_5_, 293.1384).

### Cell Culture

B16F10 melanoma and RAW 264.7 macrophage cell lines were cultured in Dulbecco's Modified Eagle Medium (DMEM; Gibco, USA) supplemented with 10% heat-inactivated fetal bovine serum (FBS; Gibco) and antibiotics. Cells were maintained at 37°C in a humidified incubator with 5% CO_2_.

### Isolation of Primary Splenocytes

Murine spleens were aseptically harvested and mechanically dissociated with a syringe plunger, then filtered through a 45-μm nylon mesh strainer. The resulting cell suspension was centrifuged at 500 × g for 3 min at 4°C. After removing the supernatant, erythrocytes were lysed by resuspending the cell pellet in 3 mL of ammonium-chloride-potassium (ACK) lysis buffer and incubating at 37°C for 3 min. Lysis was terminated by adding 6 mL of RPMI-1640 medium, and cells were centrifuged at 500 × g for 3 min at 4°C. The supernatant was discarded, and the pellet was resuspended in fresh RPMI-1640 medium for subsequent use.

### Cell Viability Assay

RAW264.7 and B16F10 cells were seeded in 24-well plates (2 × 10^4^ and 1 × 10^4^ cells/well, respectively) and incubated overnight to allow attachment. Cells were treated with twofold serial dilutions of the compounds, starting at 10 μM. After 48 h, cells were harvested and mixed with 0.4% trypan blue at a 1:1 ratio. Viable cells were counted using a hemocytometer (Marienfeld Superior, Germany).

### Melanin Content Assay

B16F10 cells (4 × 10^4^ cells/well) were seeded in 6-well plates and treated with the compounds in the presence of 100 nM a-melanocyte-stimulating hormone (α-MSH; Sigma-Aldrich, USA) for 48 h to induce melanin production. Cells were washed with phosphate-buffered saline (PBS) and lysed by adding 200 μL of 1 N NaOH containing 10% dimethyl sulfoxide (DMSO) per well. Lysates were incubated at 60°C for 1 h to solubilize melanin. Melanin content was quantified by measuring absorbance at 475 nm with a microplate reader.

### Intracellular Tyrosinase Activity Assay

B16F10 cells (4 × 10^4^ cells/well) were seeded in 6-well plates and pretreated with the compounds, then stimulated 1 h later with 100 nM α-MSH. After 48 h, cells were washed with PBS and lysed in 200 μL of 100 mM sodium phosphate buffer (pH 6.8) containing 1% Triton X-100 (Thermo Fisher Scientific, USA) per well. Lysates were centrifuged at 16,000 × g for 20 min at 4°C, and supernatants were collected. For the tyrosinase activity assay, 80 μL of the supernatant was transferred to a 96-well flat-bottom plate, and 20 μL of 10 mM L-3,4-dihydroxyphenylalanine (L-DOPA; Sigma-Aldrich), a tyrosinase substrate, was added. The reaction mixture was incubated at 37°C for 1 h, and absorbance was measured at 475 nm using a microplate reader. Tyrosinase activity was expressed as a percentage relative to the control using the following equation: Tyrosinase activity (%) = (absorbance of control/absorbance of sample) × 100.

### Quantitative Real-Time Reverse Transcription PCR (qRT-PCR)

Total RNA was extracted from cells using TRI Reagent (MRC, Cincinnati, OH), and 2 μg of total RNA was reverse-transcribed into complementary DNA (cDNA) using Moloney murine leukemia virus reverse transcriptase (M-MLV RTase; Promega, USA), oligo(dT) primers, deoxynucleotide triphosphates (dNTPs), and RT reaction buffer at 42°C for 1 h. The synthesized cDNA was combined with AccuPower^®^ 2 × GreenStar™ qPCR Master Mix (Bioneer, Republic of Korea) and 10 pmol of gene-specific primers. Quantitative real-time PCR (qRT-PCR) was performed on an Exicycler™ real-time PCR system (Bioneer) with the following cycling conditions: an initial denaturation at 95°C for 15 s, followed by 45 cycles of denaturation at 95°C for 15 s, annealing at 55°C for 15 s, and extension at 72°C for 15 s. The sequences of the primers used in qRT-PCR are as follows: GAPDH: Forward, 5′-CATCACTGCCACCCA GAAGACTG-3′ and Reverse, 5′-ATGCCAGTGAGCTTCCCGTTCAG-3′; Tyrosinase: Forward, 5′-GTACTTGGGAGGTCGTCACC-3′ and Reverse, 5′-GTCCCTCAGGTGTTCCATCG-3′; Tyrp1: Forward, 5′-GCA CACTTTCACTGATGCGG-3′ and Reverse, 5′-TAGGTGCGTTTTCCAACGGG-3′; Tyrp2: Forward, 5′-TTGCTCTTGGGGTTGCTGGCT TTTC-3′ and Reverse, 5′-TCCTCCGTGTATCTCTTGCTGCTGA-3′, IL-1β: Forward, 5′-ACCTGTGTCTTTCCCGTGG-3′ and Reverse, 5′-TCATCTCGGAGCCTGTAGTG-3′, IL-6: Forward 5′-AGTTGTGCAATGGCAATTCTGA-3′ and Reverse, 5′-AGGACTCTGGCTTTGTC TTTCT-3′, TGF-β: Forward, 5′-TGACGTCACTGGAGTTGTACGG-3′ and Reverse, 5′-GGTTCATGTCATGGATGGTGC-3′, IL-4: Forward, 5′-ACAGGAGAAGGGACGCCAT-3′ and Reverse, 5′- GAAGCCCTACAGACGAGCTCA-3′.

### Enzyme-Linked Immunosorbent Assay (ELISA)

To evaluate the anti-inflammatory and anti-melanogenic effects of the HC mix, various ELISA protocols were performed. For cytokine analysis, RAW 264.7 cells were seeded in 24-well plates at a density of 1 × 10^4^ cells/well. After 1 h of pretreatment with the HC mix, cells were stimulated with 100 ng/mL lipopolysaccharide (LPS; Sigma-Aldrich) for 48 h. Culture supernatants were harvested, and the levels of IL-1β (Cat No. DY401), IL-6 (Cat No. DY406), and TGF-β (Cat No. DY1679) were quantified using commercial ELISA kits (R&D Systems, USA). To determine intracellular inducible nitric oxide synthase (iNOS) expression, RAW 264.7 cells were pretreated with the HC mix for 1 h and stimulated with LPS (100 ng/mL) for 24 h. Following lysis, iNOS expression in the cell lysates was quantified using an iNOS-specific ELISA kit (Cat No. ab253219; Abcam, UK).

For melanogenic protein expression, B16F10 cells were seeded at 4 × 10^4^ cells/well and pretreated with the HC mix for 1 h, followed by stimulation with 100 nM α-MSH for 48 h. After washing and lysis with RIPA buffer, total protein concentrations were determined via BCA assay (Thermo Fisher Scientific, USA). Lysates were diluted to 5 μg/mL in carbonate-bicarbonate coating buffer (pH 9.6) and coated onto 96-well microplates at 4°C overnight. The plates were blocked with 1% bovine serum albumin (BSA) and incubated with primary antibodies against Tyrosinase (sc-20035), Tyrp1 (sc-166857), and Tyrp2 (sc-74439; Santa Cruz Biotechnology, USA). After incubation with a goat anti-mouse IgG-HRP secondary antibody (sc-2005; Santa Cruz Biotechnology), immunoreactive proteins were detected using a TMB substrate. Absorbance was measured at 450 nm using a microplate reader (Molecular Devices, USA).

### Measurement of Intracellular ROS and Nitric Oxide (NO) Production

Intracellular reactive oxygen species (ROS) levels were measured using the fluorescent probe 2′,7′-dichlorodihydrofluorescein diacetate (DCF-DA; Sigma-Aldrich). Briefly, RAW 264.7 cells were seeded in 24-well plates (1 × 10^4^ cells/well) and pretreated with the HC mix for 1 h before stimulation with LPS (100 ng/mL) for 24 h. The cells were then washed with PBS and stained with 10 μM DCF-DA for 30 min at 37°C. Following an additional wash with PBS, intracellular ROS levels were analyzed using an Attune™ NxT flow cytometer (Thermo Fisher Scientific), and the resulting data were processed using FlowJo software.

Nitric oxide (NO) production was assessed by measuring nitrite concentrations in the culture supernatants using the Griess reaction. Briefly, supernatants were collected 24 h after LPS stimulation and mixed with an equal volume of Griess reagent (1% sulfanilamide in 5% phosphoric acid, and 0.1% N-(1-naphthyl)ethylenediamine dihydrochloride; all reagents were purchased from Sigma-Aldrich). After incubation at room temperature for 10 min, the absorbance was measured at 540 nm using a microplate reader (Molecular Devices). Nitrite levels were quantified based on a standard curve generated using sodium nitrite.

### Statistical Analysis

All experiments were performed in at least five independent biological replicates. Data are presented as the mean ± standard deviation (SD). Statistical significance between groups was determined by one-way analysis of variance (ANOVA), followed by Tukey’s post-hoc test for multiple comparisons. A p-value of less than 0.05 was considered statistically significant. All statistical analyses were conducted using GraphPad Prism 11.01 (GraphPad Software, USA).

## Results

### Identification based on morphological characteristics and phylogenetic analysis.

Colonies grew slowly on PDA, reaching 33 mm in diameter after 7 days at 25°C, slightly raised at the center; mycelium white and yellow; texture velvety and floccose; sporulation absent; reverse olivaceous to citrine (Supplementary Fig. S1 A, D, G). The microscopic features of *Curvularia intermedia* CNUFC 23CHB15 are presented in Supplementary Fig. S1H and I, showing the hyphae and coiled hyphae. Despite repeated attempts, sporulation could not be induced under the tested growth conditions. However, phylogenetic analyses clearly placed strain CNUFC 23CHB15 within *Curvularia intermedia*, and it was therefore identified as *Curvularia intermedia*. The multigene analysis included 27 taxa, with Bipolaris maydis CBS 136.29 as the outgroup. The concatenated alignment consisted of 2074 characters (including alignment gaps): 634, 549, and 891 for ITS, *GAPDH*, and *TEF1*, respectively. Analysis of the combined dataset of ITS, *GAPDH*, and *TEF1* sequences revealed that CNUFC 23CHB15 clustered together with *Curvularia intermedia* (UTHSC:08-1041, CBS 334.64, and JSNJ-2019) (Supplementary Fig. S2).

### Structural Characterization of Curvularin Derivatives (compounds 1-3).

Preliminary HRESI-LC-MS/MS analysis of the *Curvularia intermedia* extract revealed that curvularin and its derivatives were the predominant constituents. To evaluate the anti-melanogenic potential of metabolites derived from *C. intermedia*, the ethyl acetate extract was subjected to reversed-phase C18 column chromatography using an acetonitrile-water gradient (containing 0.1% formic acid). This separation yielded curvularin (compound **3**) and a 55:45 mixture of 11-hydroxycurvularin diastereomers: (11*S*,15*R*)-11-hydroxycurvularin (compound **1**) and (11*R*,15*R*)-11-hydroxycurvularin (compound **2**), hereafter referred to as the HC mix. Subsequent optimization of preparative HPLC conditions enabled the separation of compounds **1** and **2** for structural elucidation. Detailed 1D and 2D NMR spectroscopic analyses (Supplementary Figs. S3-S8), together with comparison to previously reported data, confirmed their identities as compound **1** [26], compound **2**, and compound **3** [20]. However, because compounds 1 and **2** exhibited nearly identical retention times in analytical HPLC, large-scale separation was not feasible. Therefore, in addition to the individually isolated compounds **1–3**, a 1:1 diastereomeric mixture of compounds **1** and **2** was prepared and evaluated in the anti-melanogenic assays.

### Determination of Non-Cytotoxic Concentration

**Fig. 2:**
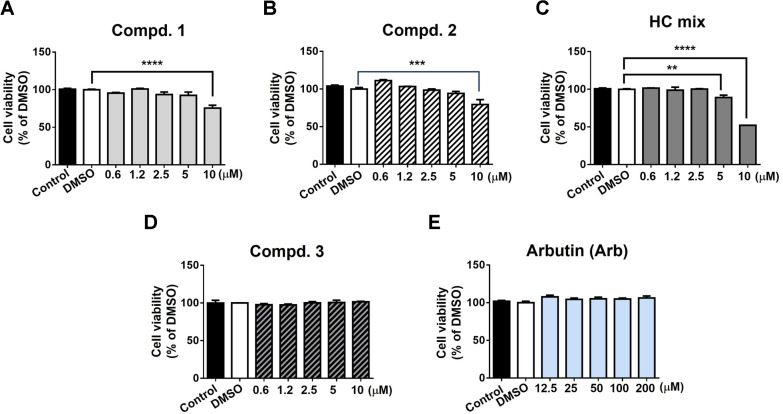
Effects of *Curvularia intermedia*-derived compounds and arbutin on the viability of B16F10 melanoma cells. B16F10 cells were treated with various concentrations (0.6–10 μM) of (**A**) compound **1**, (**B**) compound **2**, (**C**) HC mix, (**D**) compound **3**, or (**E**) Arbutin (Arb) (12.5–200 μM) for 48 h. The trypan blue exclusion assay assessed cell viability, and viable cells were counted with a hemocytometer. Data are expressed as a percentage of the DMSO-treated control group and are shown as the mean ± SD. Results are representative of five independent experiments. Statistical significance was evaluated using one-way ANOVA followed by Tukey’s post-hoc test. **p < 0.01, ***p < 0.001, and ****p < 0.0001 compared to the DMSO control.

Prior to assessing anti-melanogenic activity, the cytotoxicity of these compounds and the reference inhibitor, arbutin (Arb), was evaluated in B16F10 melanoma cells. Cells were treated with a range of concentrations (0.6−10 μM for fungal metabolites; 12.5-200 μM for Arb) for 48 h. The trypan blue exclusion assay revealed that both compounds **1** and **2** significantly reduced cell viability at 10 μM (*p* < 0.0001), while the HC mix exhibited significant cytotoxicity starting from 5 μM (*p* < 0.01) (Fig. 2A-C). In contrast, compound **3** and Arb did not significantly affect cell viability across the tested concentration ranges (Fig. 2D and 2E). Based on these results, non-cytotoxic optimal concentrations were determined to rule out the possibility that subsequent anti-melanogenic effects were due to impaired cell viability, as follows: ≤5 μM for compound **1** and compounds **2**, ≤2.5 μM for the HC mix, 10 μM for compound **3**, and 2.5 μM for Arb.

### Enhanced Anti-Melanogenic Potency of the HC Mix Compared with Its Individual Diastereomers and Curvularin

**Fig. 3:**
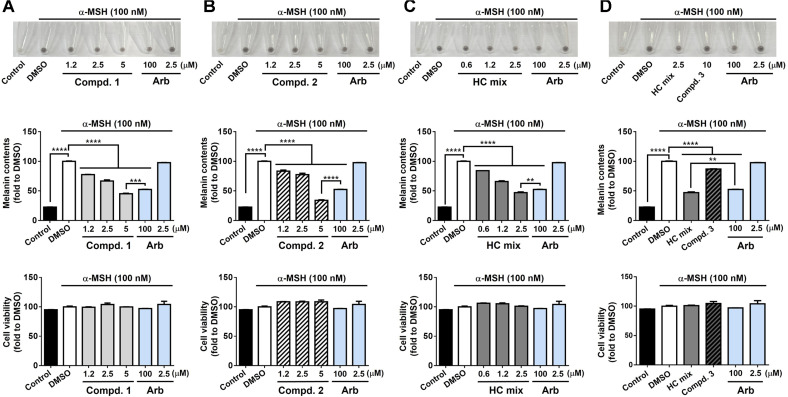
Inhibitory effects of hydroxycurvularin diastereomers, HC mix, and curvularin on α-MSH-induced melanin synthesis in B16F10 cells. B16F10 cells were treated with the indicated concentrations of (**A**) compound **1**, (**B**) compound **2**, (**C**) HC mix, or (**D**) HC mix and compound **3** in the presence of 100 nM α-MSH for 48 h. Arbutin (Arb, 100 and 2.5 μM) was used as a positive control. Upper panels: Representative photographs of B16F10 cell pellets showing visible changes in melanin pigmentation. Middle panels: Quantitative analysis of melanin content. Cells were lysed with 1 N NaOH containing 10% DMSO, and absorbance was measured at 475 nm. Lower panels: Cell viability was assessed using the trypan blue exclusion assay to confirm that the anti-melanogenic effects were not due to cytotoxicity. Data are expressed as a fold change relative to the DMSO-treated control. Data are shown as the mean ± SD values and are representative of five independent experiments. Statistical significance was evaluated using one-way ANOVA followed by Tukey’s post-hoc test. **p < 0.01, ***p < 0.001, and ****p < 0.0001 compared to the α-MSH-stimulated DMSO control.

To evaluate the inhibitory effects of *C. intermedia*-derived compounds on melanogenesis, we measured melanin production in B16F10 melanoma cells stimulated with a-melanocyte-stimulating hormone (α-MSH). α-MSH stimulation significantly increased melanin synthesis, as evidenced by the dark coloration of the cell pellets in the DMSO control group (Fig. 3). Treatment with compounds **1** and **2**, as well as their mixture, resulted in a dose-dependent lightening of the cell pellet color (upper panels) and a corresponding decrease in melanin content (Fig. 3A-3C, middle panels). Notably, at 2.5 μM, the HC mix showed greater anti-melanogenic potency (melanin content reduced to 47.1% of the DMSO control) than either compound **1** (66.7%) or compound **2** (77.4%). Compared with its structural analog compound **3**, their mixture (2.5 μM) showed a markedly greater inhibitory effect, as evidenced by visibly lighter cell pellets and reduced melanin content, even when compound **3** was used at a four-fold higher concentration (10 μM; 87% melanin content) (Fig. 3D, upper and middle panels). Parallel cell viability assays confirmed that none of the compounds induced significant cytotoxicity at the concentrations used for melanin inhibition (up to 10 μM) (lower panels). Accordingly, the observed reduction in melanin was due to inhibition of melanogenesis rather than a decrease in cell number. These data demonstrate that the HC mix exhibits enhanced anti-melanogenic activity compared with its individual diastereomers and curvularin, without impairing cell viability.

### HC Mix Inhibits Tyrosinase Activity and Melanogenic Gene Expression

**Fig. 4:**
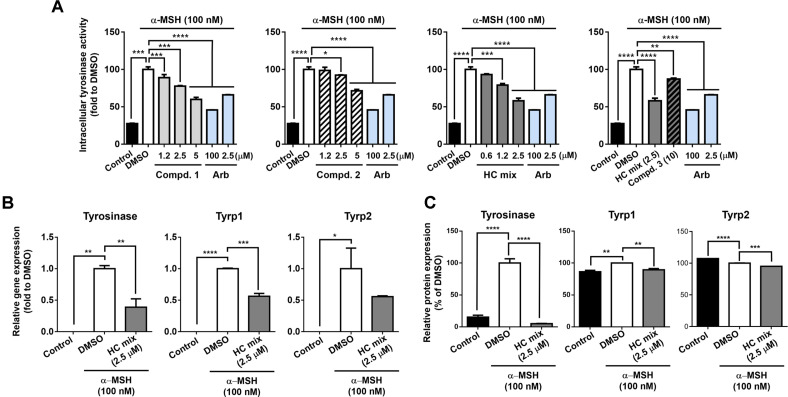
The HC mix inhibits melanogenesis by suppressing tyrosinase activity and downregulating melanogenic gene expression. (**A**) Effects of compounds **1** and **2**, HC mix, and compound **3** on intracellular tyrosinase activity in B16F10 cells. Cells were pretreated with the indicated concentrations of compounds and then stimulated with 100 nM α-MSH for 48 h. Cell lysates were incubated with 10 mM L-DOPA substrate, and tyrosinase activity was quantified by measuring absorbance at 475 nm. (**B**) Inhibitory effects of the HC mix (2.5 μM) on the mRNA expression of key melanogenic genes. After pretreatment with the HC mix and α-MSH stimulation for 24 h, total RNA was isolated, and qRT-PCR was used to analyze the relative expression levels of Tyrosinase, Tyrp1, and Tyrp2. (**C**) B16F10 cells were pretreated with the HC mix for 1 h and stimulated with 100 nM α-MSH for 48 h. The protein levels of Tyrosinase, Tyrp1, and Tyrp2 in cell lysates were determined by ELISA. Data are expressed as the mean ± SD of five independent biological replicates. Significance was determined using Tukey’s post-hoc test; *p < 0.05, **p < 0.01, ***p < 0.001, and ****p < 0.0001 indicate significant differences compared with the α-MSH-treated vehicle (DMSO) control.

Tyrosinase is the rate-limiting enzyme in melanin biosynthesis, catalyzing the oxidation of tyrosine under the transcriptional control of α-MSH [27, 28]. To investigate whether the anti-melanogenic activity of the HC mix was associated with the regulation of tyrosinase, we measured intracellular tyrosinase activity in α-MSH-stimulated B16F10 cells treated with various concentrations of the compounds. α-MSH stimulation markedly increased tyrosinase activity, which was suppressed by all tested compounds in a dose-dependent manner (Fig. 4A). Among them, the HC mix exhibited the most potent inhibitory activity. Notably, at 2.5 μM, the HC mix reduced tyrosinase activity to 58.1% of the DMSO-treated control, significantly lower than that observed for the individual diastereomers compound **1** (77.7%) and compound **2** (92.4%), as well as for the structural analog compound **3** (85.2% at 2.5 μM). Furthermore, the HC mix demonstrated inhibitory efficacy comparable to or greater than that of the positive control Arb, despite being used at a substantially lower concentration (2.5 μM HC mix vs. 100 μM Arb). We next examined whether the HC mix regulates the transcriptional expression of key melanogenic enzymes, including tyrosinase (Tyr), tyrosinase-related protein 1 (Tyrp1), and tyrosinase-related protein 2 (Tyrp2). Quantitative real-time PCR analysis showed that α-MSH-induced mRNA expression of all three genes was significantly downregulated by 2.5 μM HC mix (Fig. 4B). Specifically, Tyr, Tyrp1, and Tyrp2 transcript levels were reduced by approximately 61.2%, 43.7%, and 44.3%, respectively, compared with the α-MSH-stimulated DMSO control. As shown in Fig. 4C, the HC mix significantly downregulated the α-MSH-induced protein expression of Tyrosinase, Tyrp1, and Tyrp2 compared with the α-MSH-stimulated DMSO control. Collectively, these findings indicate that the HC mix exerts its anti-melanogenic effect through a dual mechanism: direct inhibition of tyrosinase activity and transcriptional repression of core melanogenic enzymes (Tyr, Tyrp1, and Tyrp2).

### Multifunctional Activity of HC Mix in Attenuating Oxidative Stress and Modulating Pro- and Anti-Inflammatory Cytokine Expression

**Fig. 5:**
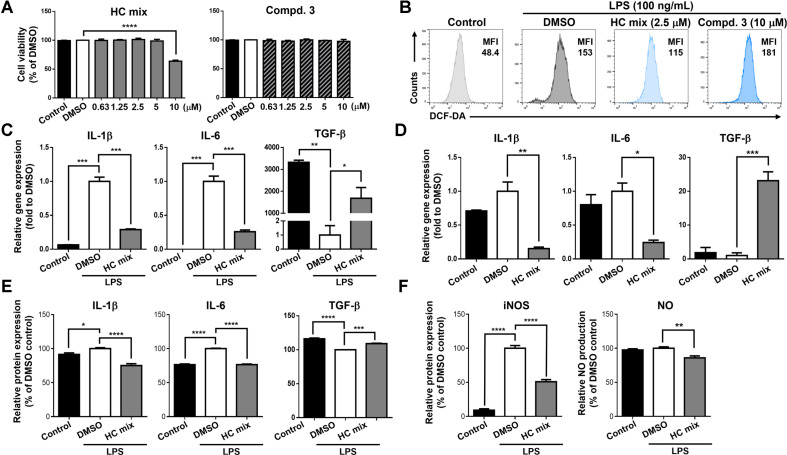
The HC mix attenuates LPS-induced oxidative stress and modulates gene expression of inflammation-related cytokines. (**A**) Effects of the HC mix and compound 3 on RAW 264.7 macrophage viability. Cells were treated with the indicated concentrations of each compound for 24 h, and viability was assessed by trypan blue exclusion. (**B**) Inhibitory effect of the HC mix on LPS-induced intracellular ROS production. RAW 264.7 cells were pretreated with the HC mix (2.5 μM) or compound **3** (10 μM) for 1 h, then stimulated with 100 ng/mL LPS for 24 h. ROS levels were measured by flow cytometry using the DCF-DA fluorescent probe. MFI denotes Mean Fluorescence Intensity. (**C, D**) Effects of the HC mix on gene and protein expression of inflammation-related cytokines in (**C**) LPS-stimulated RAW 264.7 macrophages and (**D**) unstimulated primary murine splenocytes. RAW 264.7 cells were pretreated with the HC mix (2.5 μM) for 1 h and then stimulated with LPS (100 ng/mL) for 24 h, whereas the splenocytes were treated with the HC mix alone for 24 h in the absence of LPS. mRNA expression levels of cytokines were quantified by qRT-PCR, normalized to GAPDH, and expressed relative to the vehicle (DMSO) control. (**E**) RAW 264.7 cells were pretreated with the indicated concentrations of the HC mix for 1 h and stimulated with 100 ng/mL LPS for 48 h. IL-1β, IL-6, and TGF-β protein levels in the culture supernatants were measured using ELISA. (**F**) NO production and iNOS expression in RAW 264.7 cells pretreated with the HC mix (1 h) and stimulated with 100 ng/mL LPS (24 h). NO production in supernatants was determined by the Griess reaction, and intracellular iNOS levels were quantified by ELISA. Data are represented as the mean ± SD (n = 5). Statistical significance was assessed by one-way ANOVA followed by Tukey’s post-hoc test; *p < 0.05, **p < 0.01, ***p < 0.001, and ****p < 0.0001 versus the LPS-treated vehicle (DMSO) control.

Oxidative stress and chronic inflammation are well-established drivers of dysregulated pigmentation and skin aging [29-31]. To explore the multifunctional potential of the HC mix, we investigated its antioxidant and anti-inflammatory effects in LPS-stimulated RAW 264.7 macrophages. Before assessing functional activities, we evaluated the cytotoxicity of the HC mix and its structural analog compound **3** in RAW 264.7 cells. Whereas compound **3** did not affect cell viability at concentrations up to 10 mM, the HC mix significantly reduced cell viability at 10 μM (*p* < 0.0001) (Fig. 5A). Accordingly, 2.5 μM was selected as the optimal treatment concentration for the HC mix, as it showed no detectable cytotoxicity while preserving biological activity. The antioxidant capacity of the HC mix was assessed by measuring intracellular ROS levels using DCF-DA staining. LPS treatment markedly increased ROS production (MFI: 153) compared to the untreated control (MFI: 48.4) (Fig. 5B). Treatment with 2.5 μM HC mix significantly attenuated LPS-induced oxidative stress, reducing the MFI to 115. Notably, the structural analog compound **3** (10 μM) failed to reduce ROS levels, yielding an MFI of 181, higher than that of the DMSO control. The effect of the HC mix on the expression of inflammation-related cytokines was evaluated in both LPS-stimulated RAW 264.7 macrophages and unstimulated primary murine splenocytes. Treatment of LPS-stimulated RAW 264.7 cells with the HC mix significantly suppressed LPS-induced mRNA expression of the pro-inflammatory cytokines IL-1β and IL-6, while concurrently upregulating the anti-inflammatory cytokine TGF-β (Fig. 5C). Interestingly, even without LPS stimulation, the HC mix exhibited potent immunomodulatory effects in primary murine splenocytes. Treatment of primary splenocytes with the HC mix alone significantly downregulated basal expression of the pro-inflammatory cytokines IL-1β and IL-6 and markedly upregulated expression of the anti-inflammatory cytokine TGF-β compared with the DMSO control (Fig. 5D). These findings demonstrate that the HC mix possesses robust antioxidant and immunomodulatory properties, acting through the suppression of ROS production and the balanced regulation of pro- and anti-inflammatory cytokines in both innate and adaptive immune cell models. At the protein level, the HC mix suppressed LPS-induced IL-1β and IL-6 expression while restoring TGF-β levels, which was downregulated by LPS stimulation (Fig. 5E). To further validate the anti-inflammatory efficacy of the HC mix, we examined its effect on nitric oxide (NO) production and inducible nitric oxide synthase (iNOS) expression. The HC mix significantly attenuated both LPS-induced NO production and iNOS protein expression compared with the LPS-stimulated vehicle (DMSO) control (Fig. 5F).

## Discussion

Fungal secondary metabolites are an abundant source of structurally diverse bioactive compounds with therapeutic and cosmetic potential [12, 32]. Although macrocyclic lactones such as 10,11-dehydrocurvularin have demonstrated anti-inflammatory activity [18, 33], their roles in regulating melanogenesis and immune-related pigmentation disorders remain largely unexplored. In this study, we identify a diastereomeric mixture of hydroxycurvularin (HC mix), composed of two diastereomeric polyketides derived from *Curvularia intermedia*, as a multifunctional agent with potent anti-melanogenic, antioxidant, and anti-inflammatory properties.

Our data demonstrate that the HC mix robustly inhibits α-MSH-induced melanin synthesis in B16F10 cells, with significantly greater efficacy than its individual diastereomers (compounds **1** and **2**), the parent compound curvularin (compound **3**), or the clinical comparator arbutin. This enhanced effect was observed at a lower concentration (2.5 μM), suggesting the greater inhibitory activity of the mixture (HC mix). Mechanistically, the HC mix acts via a dual mode of action: (i) inhibition of intracellular tyrosinase activity, and (ii) transcriptional repression of core melanogenic genes, including Tyr, Tyrp1, and Tyrp2. This dual regulatory mechanism contrasts with conventional depigmenting agents such as arbutin or kojic acid, which primarily target tyrosinase activity without modulating upstream gene expression. The markedly higher potency of the HC mix compared with compound **3**, despite their structural similarity, strongly suggests that hydroxylation at C-11 is a critical determinant of biological activity. This modification likely enhances the molecule's binding affinity for tyrosinase or its upstream regulatory proteins, a pattern frequently observed in the modulation of macrocyclic lactone bioactivity.

In addition to its direct anti-melanogenic effects, the HC mix showed pronounced antioxidant and anti-inflammatory activities in RAW 264.7 macrophages and primary murine splenocytes. At non-cytotoxic concentrations, it suppressed LPS-induced ROS production and modulated expression of inflammation-related cytokines. Interestingly, although the HC mix exhibited potent ROS-scavenging activity, its analog compound **3** at a higher concentration (10 μM) failed to reduce oxidative stress and even showed a slight increase in ROS levels (Fig. 5B). This disparity indicates that C-11 hydroxylation not only enhances anti-melanogenic efficacy but also mitigates potential pro-oxidant effects associated with the parent macrocyclic structure, thereby reinforcing the safety profile of the HC mix for topical use. Thus, the rationale for developing diastereomeric combinations to maximize efficacy while minimizing toxicity is crucial for the development of safe and effective bioactive formulations for skin health.

The immunomodulatory effects of the HC mix, characterized by downregulation of pro-inflammatory cytokines (IL-1β, IL-6) and upregulation of the anti-inflammatory cytokine TGF-β, are functionally significant. Oxidative stress and inflammation are now recognized not merely as secondary consequences, but as primary drivers of pigmentary dysregulation and skin aging, often referred to as “inflammaging” [34, 35]. The concurrent upregulation of TGF-β by the HC mix is particularly noteworthy. Beyond its classical anti-inflammatory role, TGF-β suppresses melanin synthesis by promoting degradation of microphthalmia-associated transcription factor (MITF), the master transcriptional regulator of melanogenesis, and by inhibiting tyrosinase activation, thereby counteracting the pro-melanogenic signaling of α-MSH [36-38]. Thus, the HC mix may establish a “non-melanogenic microenvironment” by simultaneously suppressing pro-pigmentation signals (IL-1β, IL-6) and promoting endogenous inhibitors (TGF-β). Together, the HC mix represents a potential bioactive agent that mitigates hyperpigmentation and cutaneous inflammation by reducing oxidative stress and regulating inflammatory cytokine expression.

Despite these promising findings, this study possesses several limitations. First, all experiments were conducted *in vitro* using murine melanoma and macrophage cell lines, which may not fully recapitulate the complexity of human skin physiology. Consequently, the HC mix should be considered a preliminary candidate requiring further validation in human-derived skin models or clinical studies, rather than a final cosmeceutical agent. Second, although the HC mix demonstrated potent activity, formal combination effect modeling (*e.g.*, combination index calculation) was not performed; therefore, the observed results are described as ‘enhanced’ rather than definitively synergistic. Furthermore, the precise molecular targets of hydroxycurvularin derivatives and their specific binding interactions remain to be elucidated. Future research is warranted to investigate the pharmacokinetic profiles and skin permeation potential of the HC mix, particularly regarding the interactions between the two diastereomers. Finally, subsequent studies should incorporate *in vivo* validation using animal models of hyperpigmentation and dermatitis, alongside systematic structure-activity relationship (SAR) analyses to optimize the stereochemical and functional attributes of these compounds.

In conclusion, we have identified and characterized a hydroxycurvularin mixture as a novel fungal-derived bioactive agent; however, further preclinical and human-based studies are warranted to validate its therapeutic potential. By simultaneously targeting melanogenesis, oxidative stress, and inflammation, the HC mix represents a promising therapeutic strategy for treating complex pigmentary disorders associated with skin aging and inflammation. More broadly, our findings underscore the untapped potential of fungal polyketides as a reservoir of bioactive compounds for next-generation skin therapeutics.

## Supplemental Materials

Supplementary data for this paper are available on-line only at http://jmb.or.kr.



## Figures and Tables

**Fig. 1:**
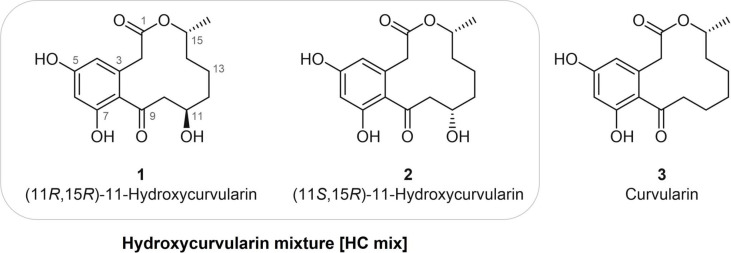
Chemical structures of curvularin and its hydroxy derivatives. Chemical structures of curvularin (compound **3**) and its two diastereomeric derivatives (compounds **1** and **2**), isolated from the fungal strain *Curvularia intermedia*. The HC mixture is a diastereomeric mixture of compounds **1** and **2**.
