# Supplementary material for: A Diastereomeric Hydroxycurvularin Mixture Inhibits Melanogenesis and Inflammation via Modulation of Tyrosinase Activity and Cytokine Expression
Source: J Microbiol Biotechnol. 2026 Jul 2;36:e2603015. doi: 10.4014/jmb.2603.03015 (PMC13372789; doi:10.4014/jmb.2603.03015)
Supplement: Supplementary file 1 [file jmb-36-e2603015-supple.pdf]

J. Microbiol. Biotechnol. 2026. 36: e2603015
https://doi.org/10.4014/jmb.2603.03015

Supplementary Figures

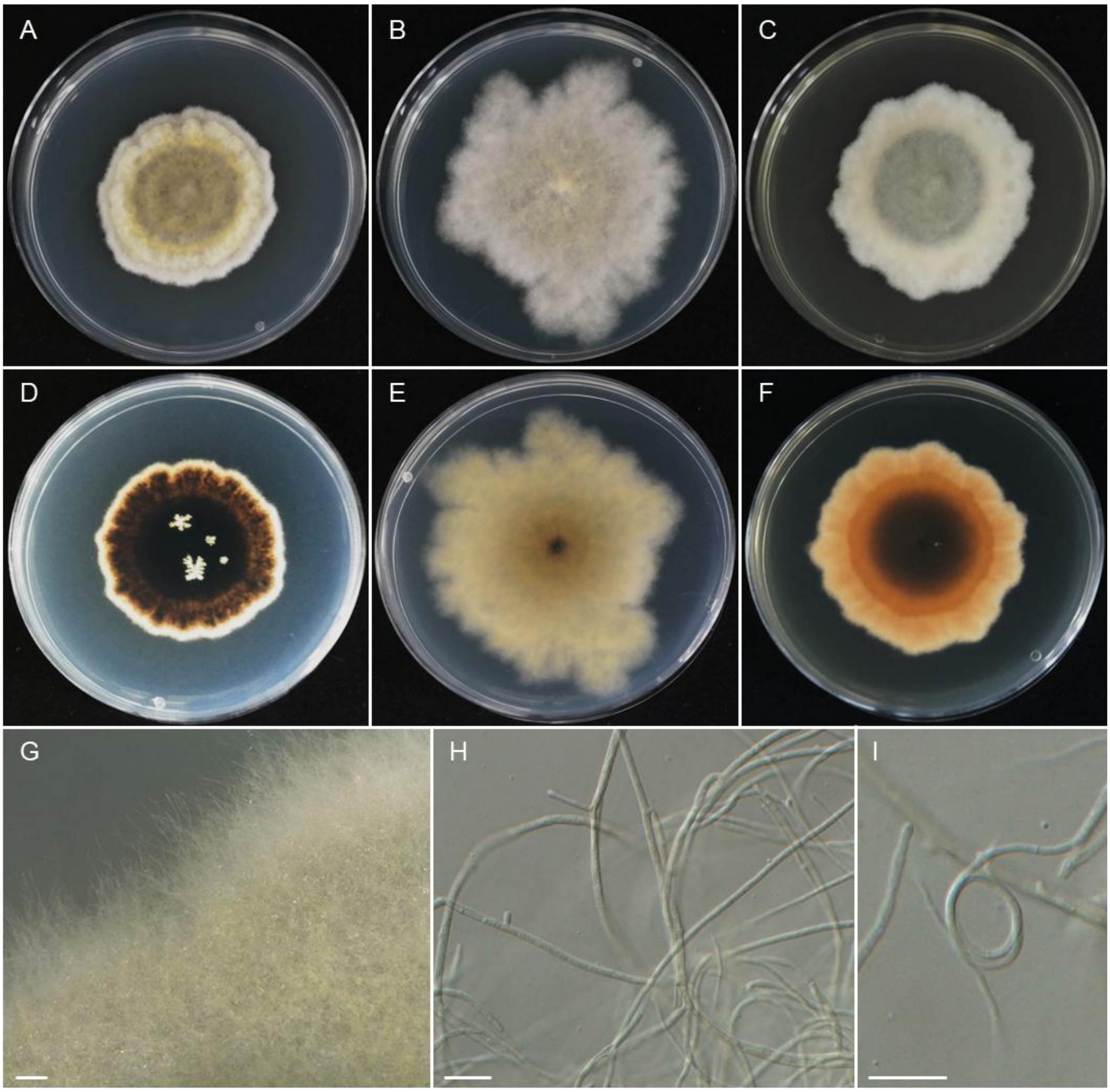

Fig. S1. ***Curvularia intermedia*** **(CNUFC 23CHB15).**

(A, D) Colony on PDA. **(B, E)** Colony on OA. (**C, F)** Colony on MEA. (**A-C**: obverse view, **D-F**: reverse view). (**G)** Colony texture on PDA. (**H, I**) Hyphae and coiled hyphae. Scale bars: **G**= 500 μm, **H, I**= 20 μm.

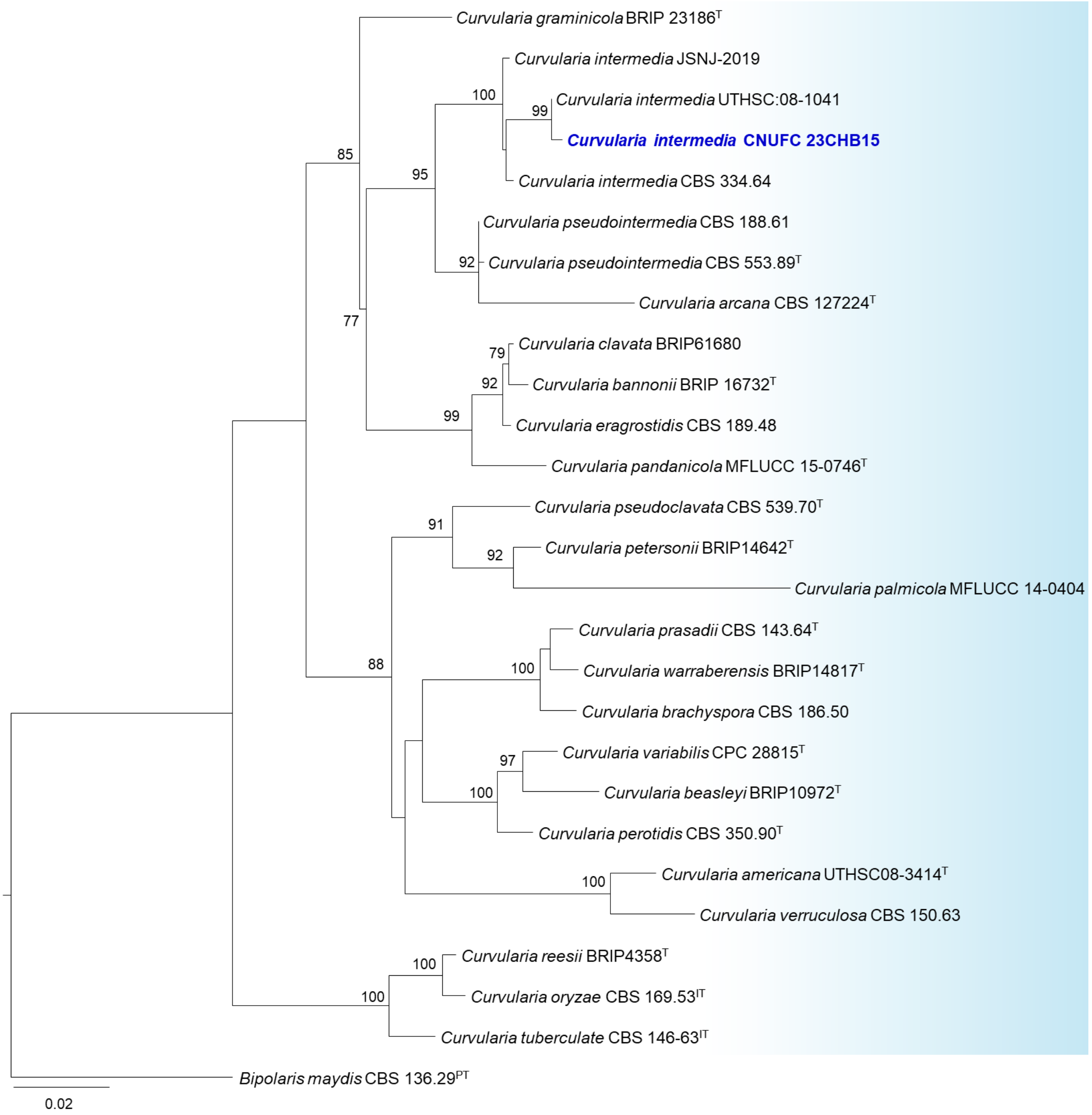

Fig. S2. Phylogram of *Curvularia* species based on combined ITS, *GAPDH*, and *TEF1* sequences.

RAxML bootstrap support values above 70% are indicated at the nodes. The tree was rooted with Bipolaris maydis CBS 136.29. The newly generated sequences are indicated in bold and blue. IT=ex-isotype, PT= ex-paratype, T= ex-type.

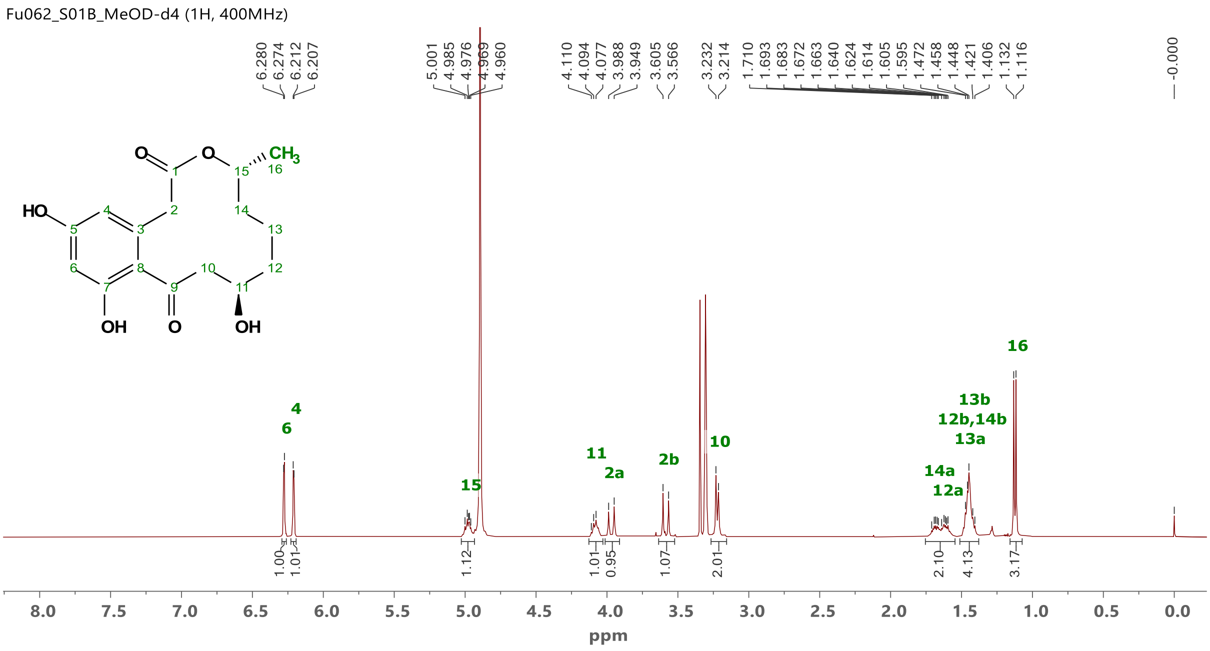

Fig. S3. ^{1}H NMR spectrum of (11*R*,15*R*)-11-hydroxycurvularin (1) in MeOH-*d4* (400 MHz).

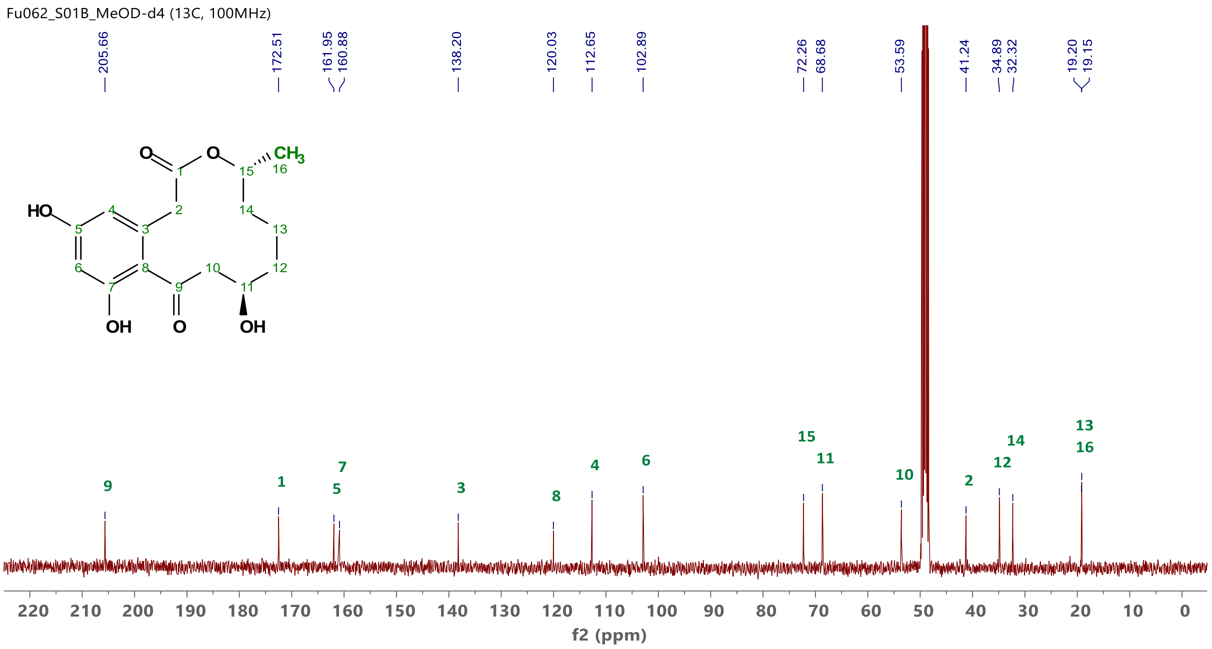

Fig. S4. ^{13}C NMR spectrum of (11*R*,15*R*)-11-hydroxycurvularin (1) in MeOH-*d4* (100 MHz).

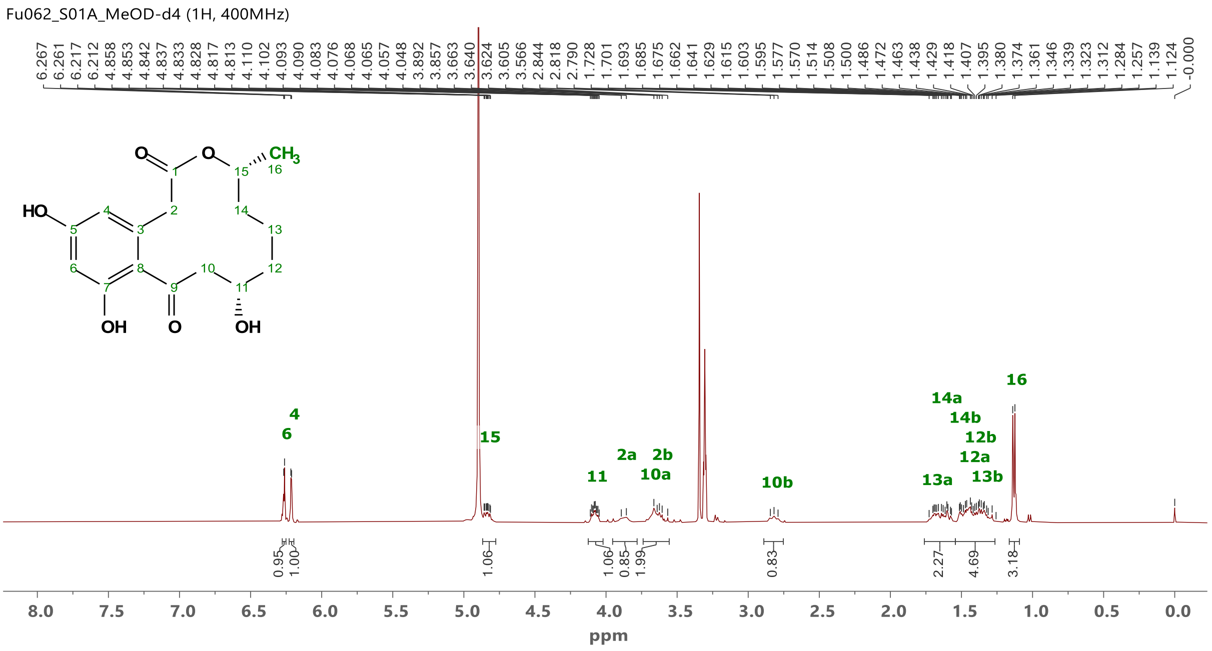

Fig. S5. ^{1}H NMR spectrum of (11*S*,15*R*)-11-hydroxycurvularin (2) in MeOH-*d4* (400 MHz).

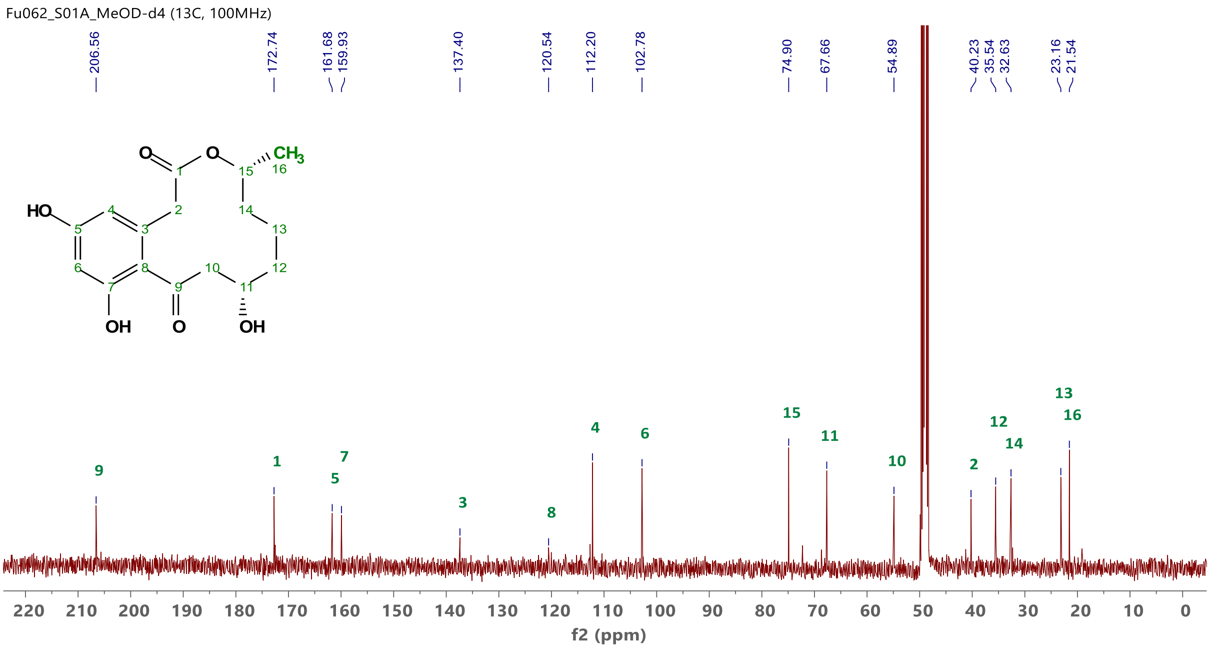

Fig. S6. ^{13}**C NMR spectrum of (11*S*,15*R*)-11-hydroxycurvularin (2) in MeOH-*d4* (100 MHz).**

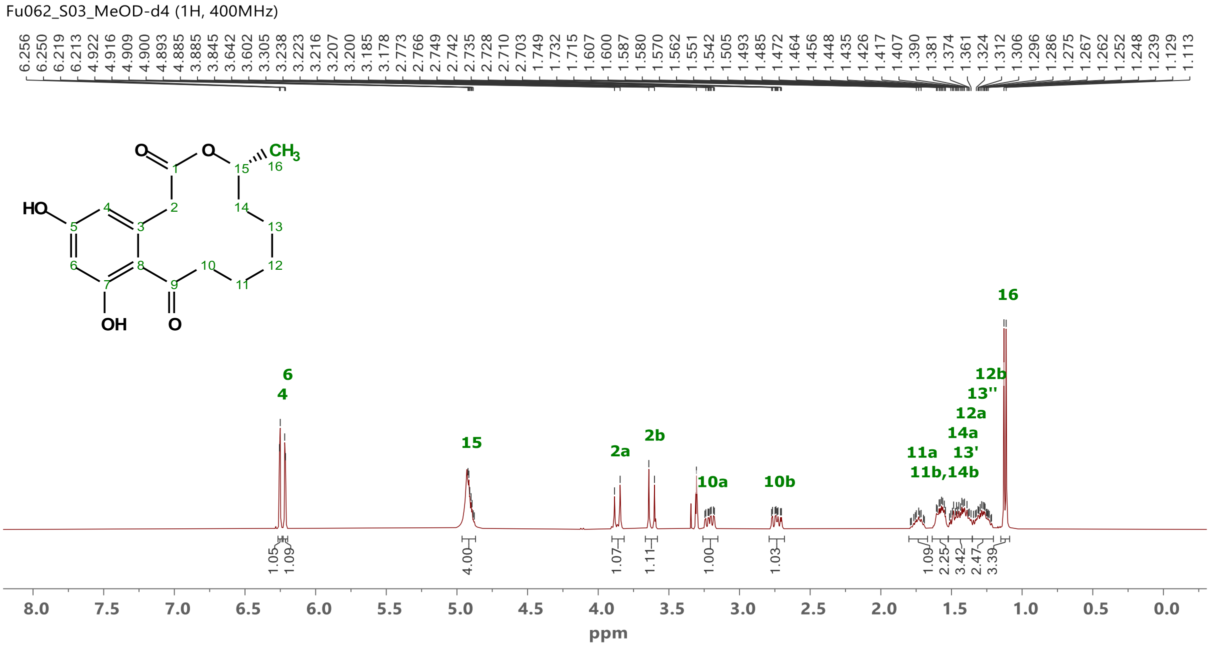

Fig. S7. ^{1}H NMR spectrum of curvularin (3) in MeOH-*d4* (400 MHz).

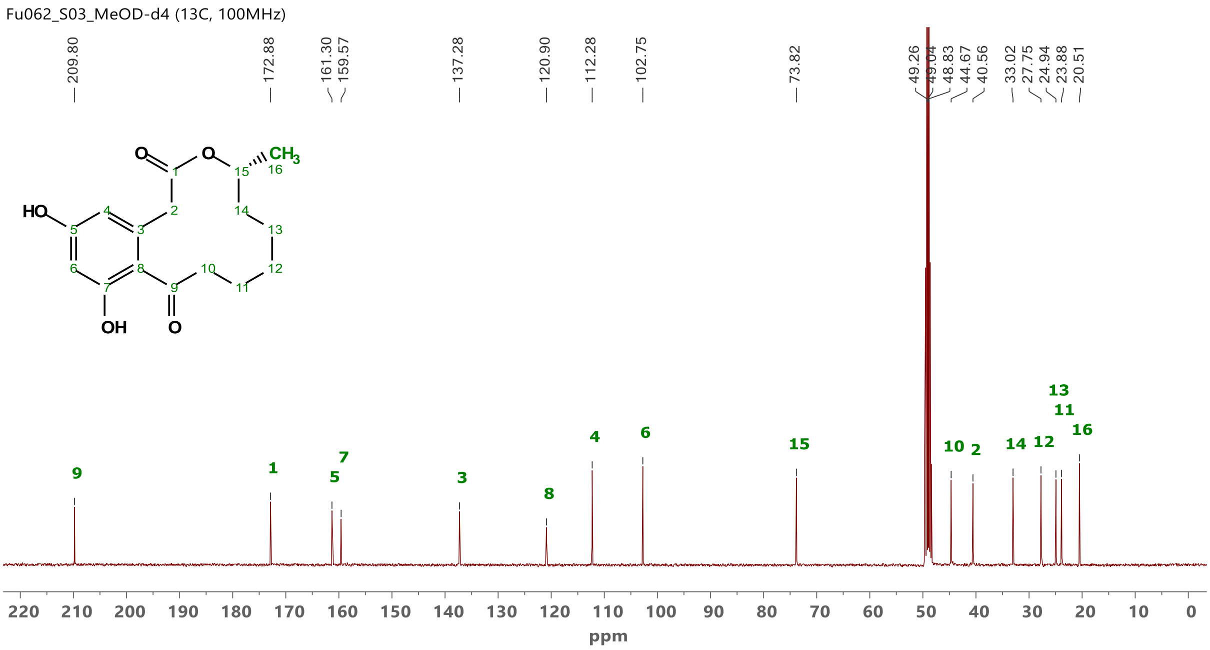

Fig. S8. ^{13}**C NMR spectrum of curvularin (3) in MeOH-*d4* (100 MHz).**
